# Prevalence, predictors, and mortality of bloodstream infections due to methicillin-resistant *Staphylococcus aureus* in patients with malignancy: systemic review and meta-analysis

**DOI:** 10.1186/s12879-021-05763-y

**Published:** 2021-01-14

**Authors:** Zhouqi Li, Hemu Zhuang, Guannan Wang, Hui Wang, Ying Dong

**Affiliations:** 1 Department of Medical Oncology, The Second Affiliated Hospital, College of Medicine, Zhejiang University, Hangzhou, Zhejiang China; 2 Department of Infectious Diseases, Sir Run Run Shaw Hospital, College of Medicine, Zhejiang University, Hangzhou, Zhejiang China; 3 Department of Integrative Oncology, Cancer Hospital & Shenzhen Hospital, Chinese Academy of Medical Sciences and Peking Union Medical College, Shenzhen, Guangdong China

**Keywords:** Methicillin-resistant *Staphylococcus aureus*, MRSA, Bacteremia, Cancer, Prevalence

## Abstract

**Background:**

Cancer patients are more likely to develop and die of bloodstream infection (BSI) than noncancer patients. Methicillin-resistant *Staphylococcus aureus* (MRSA), which is associated with immense mortality and economic burden worldwide, is not covered by the recommended initial antibiotic therapy for cancer patients with BSI. This systemic review was performed to estimate the global methicillin-resistant *Staphylococcus aureus* (MRSA) prevalence among bacteremia in patients with malignancy, and further study the predictors and mortality of cancer patients with MRSA bacteremia.

**Methods:**

The PubMed and EMBASE databases were searched for studies published from Jan. 2000 to Mar. 2020 that provided primary data on the prevalence, predictors, or mortality of MRSA bacteremia in cancer patients. A random-effects model meta-analysis was performed to estimate the pooled prevalence of MRSA with 95% confidence intervals (95% CIs).

**Results:**

The pooled prevalence of MRSA was 3% (95% CI 2–5%) among all bloodstream infections (BSIs) and 44% (95% CI 32–57%) among *S. aureus* bacteremia in cancer patients. Based on geographical stratification, the pooled prevalence was 5% in Africa (95% CI 1–14%), 1% in Americas (95% CI 1–2%), 2% in Europe (95% CI 1–4%), 4% in Western Pacific (95% CI 2–7%), 8% in South-east Asia (95% CI 4–14%) and 0% in Eastern Mediterranean (95% CI 0–3%). No significant temporal change in MRSA rates was detected in this analysis (*R*^2^ = 0.06; *P* = 0.24). Predictors for MRSA BSIs among cancer patients were identified by comparison with their methicillin-susceptible counterparts, and they were mainly related to healthcare-associated infections and immunosuppression. Finally, the 60-day mortality in adult cancer patients with MRSA BSIs was reported to be 12%, and the 6-month overall mortality was 43.2%, with community-onset infection, secondary BSI, and vancomycin MIC≥2 g/mL being the risk factors for mortality.

**Conclusions:**

Although the prevalence of MRSA BSIs among cancer patients is relatively low, it did not decline over time as MRSA BSIs in the general hospital population and the high mortality rate was related to MRSA BSIs in patients with malignancy.

**Supplementary Information:**

The online version contains supplementary material available at 10.1186/s12879-021-05763-y.

## Background

Cancer patients are highly susceptible to bloodstream infection (BSI) due to frequent hospital admissions, cytotoxic chemotherapy, use of invasive procedures, and exposure to broad-spectrum antibiotics [1, 2]. Accordingly, they witnessed a more significant increase in the incidence of BSI, and a higher mortality rate than noncancer patients in recent years [3], with prevalence ranging from 11 to 38% and the mortality rate around 40% [4]. In a study investigating nearly 14 million patients with BSI in the US from 2006 to 2014, Gram-positive bacteria are found to be the leading causative pathogens (27.38%) in cancer patients [3]. despite a shift from Gram-positive to Gram-negative organisms has been documented in recent years [5].

*Staphylococcus aureus*, a common Gram-positive bacterium colonizing the skin, the nares, and the perineum, frequently causes skin, soft tissue, and bloodstream infections in human beings [6]. *S. aureus* Bacteremia (SAB) is one of the most serious situations in *S. aureus* infections and is related to mortality rates of 15–60% [7, 8]. Underlying malignancy has been reported to be a risk factor for mortality in patients with SAB in several studies [9–12]. In turn, the presence of SAB also indicated an increased risk of death in cancer patients [13, 14].

*S. aureus* is known to be frequently antibiotic-resistant, and methicillin-resistant *S. aureus* (MRSA) infections have been the main cause of mortality and the immense economic burden attributed to *S. aureus* infections worldwide [15, 16]. Methicillin-resistance adversely affected the outcome of patients with SAB whether in the general population or cancer patients [9, 14, 17–19], and appropriate empirical antibiotic treatment significantly improved the outcome [20]. The recommended first-line therapy for MRSA bacteremia is appropriately dosed vancomycin, with daptomycin an effective alternative [21]. However, the NCCN Guidelines strongly recommended vancomycin not be routinely included in the empiric therapy alone for cancer patients [22], which could impair the prognosis of patients with MRSA BSI. Thus, there is an urgent need for data on the prevalence, and the risk factors for the development and mortality of MRSA BSIs in cancer patients for better management of MRSA in this population. In this systemic review, we performed a meta-analysis on the global MRSA prevalence among bacteremia in patients with malignancy and further summarized the limited information on the predictors and mortality of cancer patients with MRSA BSI.

## Methods

This study was conducted and reported based on the Preferred Reporting Items for Systematic Reviews and Meta-Analyses (PRISMA) guidelines [23].

### Literature search

We searched the PubMed and EMBASE databases for studies published up to 11 Mar 2020. The search strategy consisted of #1 (tumor* OR tumour* OR cancer* OR carcinoma* OR malignanc* OR neoplasia* OR oncolog* OR sarcoma* OR hematolog* OR haematolog* OR leukemia* OR leukaemia* OR lymphoma*), #2 (“bloodstream infection*” OR “blood stream infection*” OR bacteremia OR bacteraemia), #3 (“Methicillin-Resistant *Staphylococcus aureus*” OR MRSA OR “*Staphylococcus aureus*” OR “*S. aureus*”). The search was conducted as #1 AND #2 AND #3 limited to the title and abstract with the filters: human, English, and recent 20 years. Additional articles were identified by manually searching reference lists of the identified articles and relevant reviews [24, 25]. Further articles citing the included studies were retrieved using Web of Science.

### Study selection

Studies were included if they met either inclusion criterion, and did not meet any of the exclusion criteria. The inclusion criteria were: 1. studies containing primary data on the total number of blood isolates from BSI cases and the number of MRSA isolates in 10 or more cancer patients; 2. studies that provided data on the predictors or mortality of cancer patients with MRSA BSI. The exclusion criteria were: 1. no differentiation between laboratory-confirmed infections and contamination; 2. studies that focused on microbial isolates with no relation to clinical BSI cases; 3. MRSA outbreak; 4. non-English publications; 5. not available in full-text, including conference abstracts. Title and abstract screening and full-text assessment were conducted independently by two authors after duplicates were removed, with discrepancies resolved by consensus.

### Data extraction and quality assessment

Data collected included study characteristics (primary author, year of publication, country, study design, study period, and study setting), prevalence (the total number of BSI isolates and the number of isolates of Gram-positive bacteria, *S. aureus*, and MRSA). The total number of microbial isolates from cancer patients with BSI was collected to calculate the prevalence of MRSA, because the number of patients, as well as the BSI episodes, was not available in all studies. MRSA rates in *S. aureus* were calculated in a study when the number of *S. aureus* was more than 10. Furthermore, demographic and clinical factors associated with MRSA predictors and mortality, as well as the mortality rate of patients with MRSA BSIs, were collected.

The methodological quality of all identified studies was evaluated by the Newcastle-Ottawa scale [26]. Based on this scale, studies received stars across three categories: selection and comparability of study groups, and outcome ascertainment. Generally, the rating criteria were: Low quality = 0–5; medium quality = 6–7; and high quality = 8–9. The studies on prevalence could score five stars at most and were considered of high quality with four or five stars [24], because the non-exposed cohort and the outcome at the beginning were not applicable to these studies.

### Statistical analysis

A random-effects meta-analysis was performed to estimate the pooled prevalence of MRSA with 95% confidence intervals (95% CIs) [27]. World Health Organization (WHO) geographical stratification was applied to study the geographic variation of MRSA prevalence. Temporal trends in MRSA prevalence was assessed by linear regression. The proportions of MRSA isolates in two subgroups were compared with Chi-square test. The meta-analysis, linear regression, and Chi-square test were conducted by MedCalc statistical software, version 15.2 (MedCalc Software, Ostend, Belgium). Publication bias was tested by funnel plot and Egger’s test, and the data were analyzed by the statistical software R (Version 3.6.3, R Foundation for Statistical Computing, Vienna, Austria) with the meta package (Version 4.11–0). Rates in each study were stabilized using the Freeman-Tukey double-arcsine transformation before analysis to minimize the effect of extremely small rates [28]. Study heterogeneity was assessed with the I^2^ test and interpreted as relevant if > 50%. Generally, a *P*-value of < 0.05 was considered statistically significant, but statistically significant publication bias was suggested when *P-*value was less than 0.1 on Egger’s test.

## Results

### Search results, quality assessment, and studies characteristics

**Fig. 1:**
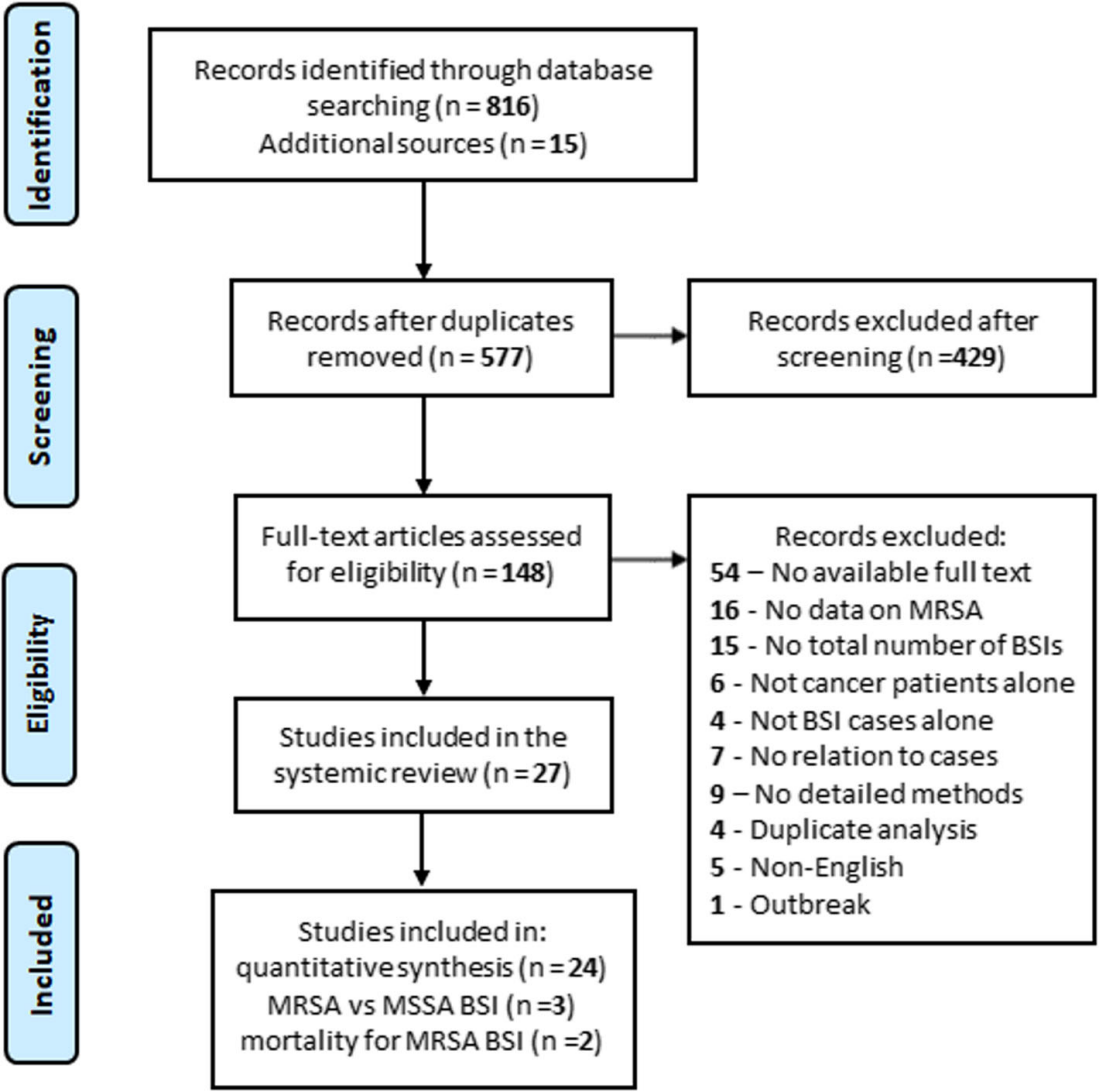
Flow chart of study selection. MRSA, methicillin-resistant *Staphylococcus aureus*; MSSA, methicillin-susceptible *Staphylococcus aureus*; BSI, bloodstream infection

As shown in Fig. 1, a total of 831 articles were retrieved from PubMed and EMBASE together with additional sources. 577 records were screened by title and abstract following the removal of duplicates, and 429 articles were crossed out. After a full-text review of the remaining 148 articles, 121 articles were excluded. Finally, 27 studies were considered eligible, with 24 included in the meta-analysis for MRSA prevalence [5, 13, 29–50], three in the potential MRSA predictors [13, 51, 52], and two in the risk factors for mortality in patients with MRSA BSIs [53]. The selected studies scored five or six according to the Newcastle-Ottawa scale and were considered of high or moderate quality (Supplementary Table S1).

The characteristics of the studies concerning MRSA prevalence are shown in Table 1. The studies collected BSI data in cancer patients between 1995 and 2017 and were from 19 countries covering all the six WHO geographical stratifications. All studies were observational; 12 were retrospective cohorts, 10 were prospective cohorts, and two were cross-sectional studies. Most of the studies were single-center studies except for two multicenter studies. More than half of the studies included only adults with a proportion of males varying between 39.7 and 69.9%. In total, 10,550 BSI isolates were included in the meta-analysis for MRSA prevalence. The characteristics of the studies included in the analysis of MRSA predictors and mortality were summarized in Supplementary Table S2.

**Table 1:** Characteristics of the included studies on MRSA prevalence

Region/Study	Country	Study perioid	Setting	No. of BSI isolates	No. of GP bacteria (%)	No. of MRSA (%)	MRSA rates in *S. aureus*
*Africa*							
El-Mahallawy et al. 2005 [43]	Egypt	1999	Children with ST or HM	328	168 (51.2)	18 (5.5)	40.9%
Arega et al. 2018 [49]	Ethiopia	2011–2012	Adults with ST or HM	82	43 (52.4)	23 (28.0)	74.2%
Obeng-Nkrumah et al. 2015 [41]	Ghana	2010–2013	Adults and children with ST or HM	93	40 (43.0)	0 (0.0)	NA
Mvalo et al. 2018 [38]	South Africa	2012–2014	Children with ST or HM	173	85 (49.1)	2 (1.2)	NA
Lubwama et al. 2019 [30]	Uganda	2014	Adults and children with ST or HM	33	11 (33.3)	1 (3.0)	NA
*Americas*							
Velasco et al. 2004 [35]	Brazil	2000–2002	Adults and children with ST or HM	1036	328 (31.7)	17 (1.6)	18.7%
Islas-Munoz et al. 2018 [5]	Mexico	2016–2017	Adults with ST or HM	496	135 (27.2)	4 (0.8)	8.0%
*Europe*							
Anatoliotaki et al. 2004 [42]	Greece	1995–2000	Adults with ST	157	54 (34.4)	12 (7.6)	85.7%
Schelenz et al. 2013 [48]	UK	1997–2010	Adults with ST or HM	949	560 (59.0)	26 (2.7)	32.1%
Miedema et al. 2013 [34]	Netherlands; Switzerland	2004–2011	Children with ST or HM and FN	248	180 (72.6)	0 (0.0)	NA
Horasan et al. 2011 [36]	Turkey	2004–2009	Adults with ST or HM and FN	98	47 (48.0)	3 (3.1)	NA
Kara et al. 2015 [46]	Turkey	2005–2009	Adults with HM and FN	536	192 (35.8)	7 (1.3)	23.3%
Bodro et al. 2014 [47]	Spain	2006–2011	Adults with ST or HM	1148	NA	13 (1.1)	12.3%
Gedik et al. 2014 [40]	Turkey	2010–2012	Adults with HM and FN	90	17 (18.9)	4 (4.4)	NA
*Western Pacific*							
Lai et al. 2003 [31]	Taiwan, China	1999	Children with ST or HM and FN	46	11 (23.9)	3 (6.5)	NA
Wang et al. 2005 [33]	Taiwan, China	1999–2002	Adults with HM	418	87 (20.8)	39 (9.3)	73.6%
Huang et al. 2011 [29]	Taiwan, China	2003–2005	Adults with ST or HM	588	153 (26.0)	24 (4.1)	55.8%
Baskaran et al. 2007 [32]	Malaysia	2004–2005	Adults with HM and FN	73	29 (39.7)	0 (0.0)	NA
Yamamoto et al. 2010 [39]	Japan	2003–2007	Adults with HM	119	89 (74.8)	14 (11.8)	82.4%
Kang et al. 2012 [13]	South Korea	2006–2007; 2008–2009	Adults with ST or HM	1246	408 (32.7)	59 (4.7)	48.4%
Kwon et al. 2013 [45]	South Korea	2009–2010	Adults with HM	243	122 (50.2)	7 (2.9)	63.6%
Chen et al. 2017 [44]	Taiwan, China	2008–2013	Adults with HM	2090	841 (40.2)	24 (1.1)	42.1%
*South-east Asia*							
Bhat et al. 2016 [37]	India	2012–2014	Adults with ST or HM and FN	128	51 (39.8)	10 (7.8)	31.3%
*Eastern Mediterranean*							
Greenberg et al. 2005 [50]	Israel	1998–2002	Children with ST or HM and FN	132	39 (29.5)	0 (0.0)	NA

*BSI* bloodstream infection, *GP* Gram-positive, *MRSA* methicillin-resistant *Staphylococcus aureus*, *ST* solid tumors, *HM* hematological malignancy, *FN* febrile neutropenia, *NA* not applicable

### Prevalence of MRSA-BSIs

**Fig. 2:**
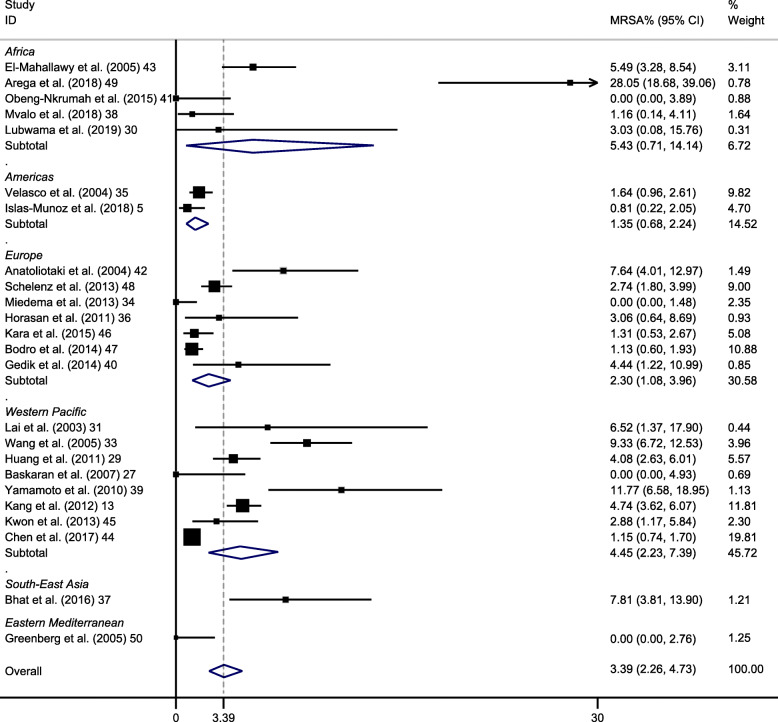
Forest plot of included studies on methicillin-resistant *Staphylococcus aureus* (MRSA) isolation rates in bacteremia among cancer patients, stratified by World Health Organization geographic regions. CI, confidence interval

Overall, the 24 data sets presented a pooled MRSA rate of 3% (95% CI 2–5%) (Fig. 2). High heterogeneity (I^2^ = 91%) was present among the studies. The funnel plot seemed approximately symmetrical (Supplementary Fig. S1) and no significant publication bias was detected by further Egger’s test (*p  =*  0.12).

Among a subset of 23 studies that contained data on 3690 Gram-positive BSI cases, the MRSA isolation rate in Gram-positive bacteria was 10% (95% CI 6–14%) [5, 13, 29–46, 48–50]. In 15 studies whose number of *S. aureus* was more than 10, the pooled prevalence of MRSA among *S. aureus* was 44% (95% CI 32–57%) [5, 13, 29, 33, 35, 37, 39, 42–49].

**Fig. 3:**
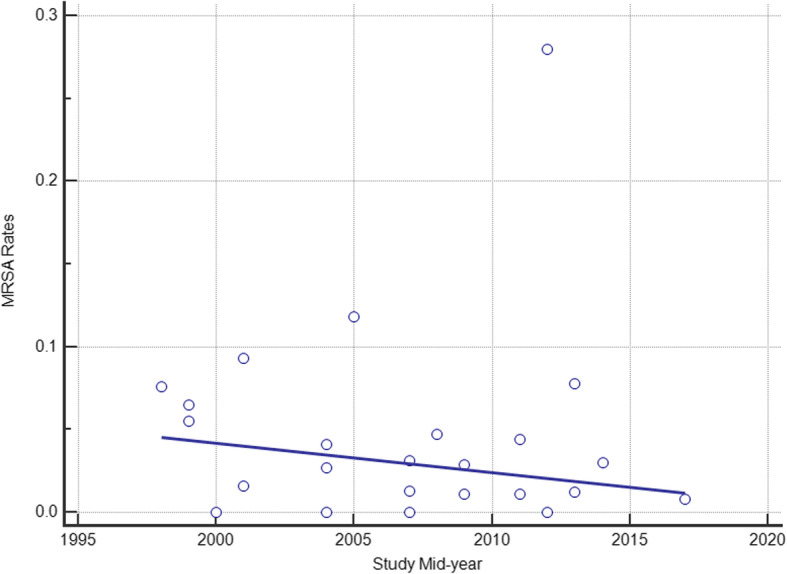
Temporal trends of MRSA isolation rates in bacteremia among cancer patients. Circles represent the prevalence from the included study and the regression line is shown by study mid-year

Subgroup analyses of MRSA prevalence were conducted by region, age group, type of cancer, and the presence of febrile neutropenia. The prevalence of MRSA was 5% in Africa (95% CI 1–14%), 1% in Americas (95% CI 1–2%), 2% in Europe (95% CI 1–4%), 4% in Western Pacific (95% CI 2–7%), 8% in South-east Asia (95% CI 4–14%) and 0% in Eastern Mediterranean (95% CI 0–3%). All 6 regions showed significantly different MRSA rates when compared to the rest of the regions (all *P* < 0.05). Ghana, an African country, saw the greatest MRSA rate (28%) which was nearly 10 times higher than the overall prevalence [49]. On the contrary, four studies from different regions (Africa, Europe, Western Pacific, and Eastern Mediterranean) did not detect MRSA in BSI cases [32, 34, 41, 50]. In addition, studies demonstrate no statistically significant changes through the examination of the temporal trend of the MRSA prevalence rates (*R*^2^ = 0.06; *P* = 0.24) (Fig. 3).

Based on data from 16 studies on 8461 adult BSI isolates, the MRSA isolation rate among BSIs in adults was 4% (95% CI 3–6%) [5, 13, 29, 32, 33, 36, 37, 39, 40, 42, 44–49]. Besides, five studies reported data on 927 BSI isolates from children and the MRSA prevalence in this population was 2% (95% CI 0–5%) [31, 34, 38, 43, 50]. When comparing the two groups, the difference in the MRSA rates was statistically significant (χ^2^ = 12.7; *P* = 0.0004).

To discriminate the MRSA prevalence in BSI cases with hematological malignancy and solid tumors, a Spanish study [54] which was previously excluded for duplicate analysis was retrieved and included in the latter group. In this way, 685 BSI cases among patients with solid tumors from two studies were identified, and the prevalence of MRSA in this subgroup was 4% (95% CI 0–12%) [42, 54]. Seven studies provided data on 3569 BSI isolates from patients with hematological malignancies and the pooled prevalence of MRSA was 4% (95% CI 1–7%) [32, 33, 39, 40, 44–46]. The difference between the two groups did not reach statistical significance (χ^2^ = 0.03; *P* = 0.86).

In the specific subgroup of patients with febrile neutropenia, a total of 1351 BSI isolates from eight studies saw a pooled MRSA rate of 2% (95% CI 1–5%) [31, 32, 34, 36, 37, 40, 46, 50]. Half of the studies were conducted in Europe [34, 36, 40, 46], two studies in the Western Pacific [31, 32], one in South-East Asia [37], and one in the Eastern Mediterranean [50].

### Predictors for MRSA-BSIs

Risk factors for the development of MRSA bacteremia among cancer patients with BSIs were summarized in Table 2. All the three relevant studies [13, 51, 52] compared the demographic and clinical characteristics of cancer patients with MRSA BSIs to their counterparts with methicillin-susceptible *S. aureus* (MSSA) BSIs. Hospital-acquired BSI was found to be more prevalent during MRSA BSIs than MSSA BSIs in two studies, although with slightly different definitions [13, 52]. Consistently, Indwelling urinary catheter, the presence of nasogastric tube, the need for catheter removal, and healthcare-associated pneumonia as origin were shown to be related to a higher possibility for MRSA development [13, 51]. In the specific population with hematologic malignancies, hospital-acquired infection was also identified as a predisposing factor for MRSA, and primary bacteremia was found to be a protective factor [52].

**Table 2:** Predictors for BSI due to MRSA in cancer patients

Study	Type of analysis	Predictor	Prevalence (%)	OR (95% CI)	P
Bello-Chavolla et al. 2018 [52]	Multivariate analysis	Hospital-acquired infection	56 (58.9)	5.54 (3.27–9.38)	< 0.001
		Healthcare-associated pneumonia	19 (20.0)	3.02 (1.63–5.59)	< 0.001
		Diabetes mellitus	67 (18.9)	2.09 (1.02–4.28)	0.049
Kang et al. 2012 [13]	Univariate analysis	Nosocomial acquisition	45 (76.3)	3.11 (1.43–6.77)	0.004
		Indwelling urinary catheter	25 (42.4)	3.90 (1.67–9.12)	0.001
		Nasogastric tube	12 (20.3)	5.11 (1.36–19.14)	0.009
		ICU admission	17 (28.8)	4.70 (1.61–13.73)	0.003
Srinivasan et al. 2010 [51]	Chi-square test	Persistently positive blood cultures	5 (50)		0.004
		Catheter removal	6 (60)		0.003

*BSI* bloodstream infections, *MRSA* Methicillin-resistant *Staphylococcus aureus*, *OR* odds ratio, *CI* confidence interval, *ICU* intensive care unit

### Mortality of MRSA-BSIs

The 60-day mortality in adult cancer patients with MRSA BSIs was reported to be 12% [53], and the 6-month overall mortality was 43.2% [52]. In those who were treated with vancomycin, the treatment failure rate was 52% with the failure defined as death, uncontrolled infection, rapid relapse, or severe adverse events [53]. Concerning the risk factors for mortality in cancer patients with MRSA BSIs, multivariate analysis revealed that community-onset infection, secondary BSI, and vancomycin MIC≥2 g/mL were significantly related to 60-day mortality [53].

## Discussion

*S. aureus* is one of the ‘ESKAPE’ organisms that are responsible for the majority of bacterial infections in patients with malignancy [55]. Besides, bacteremia and multi-drug resistance has become a growing issue in cancer patients [4]. In our study, MRSA is the causative pathogen in 3% of BSIs among patients with malignancy in general. This rate can be as high as 28% in an African country, rendering Africa being the second-highest region considering MRSA rates. However, three in five African countries had MRSA rates lower than the global pooled prevalence, indicating high heterogeneity among different regions. Statistically, high heterogeneity was present in each subgroup. Thus, The treatment of BSIs in patients with cancer should take the local microbiology and antibiotic-sensitivity patterns into account, besides referring to the established guidelines [56]. It is notable that limited information is available in the literature on MRSA BSIs in South-East Asia, Eastern Mediterranean, and Americas, suggesting the need for further studies of high quality in these regions to better understand the overall burden of MRSA BSIs in cancer patients.

In recent years, a variety of effective measures, including improvements in preventing healthcare-associated infections and MRSA transmission interruption in hospitals, were taken to control infections [57]. As a result, hospital-onset MRSA bacteremia rates substantially reduced between 2005 and 2012, and the rate of decrease has slowed since 2012 in the general population of the US [57]. However, this decline was not detected in MRSA-BSIs among patients with malignancy, which might be explained by two possible reasons: firstly, although with the emergence of colony-stimulating factors, Immunomodulatory drugs, etc., cancer patients are still exposed to cytotoxic chemotherapy, broad-spectrum antibiotics, and undergo frequent invasive procedures, rendering them immunosuppressed and susceptible to MRSA; secondly, the rate of community-associated MRSA (CA-MRSA) remains stable, and there is a surge of MRSA in some regions [16, 57]. CA-MRSA, unlike hospital-acquired MRSA which could be effectively controlled by vigorous hand hygiene, antimicrobial stewardship, and barrier precautions, lacks easily targeted prevention strategies [58]. Strains of CA-MRSA are more likely to spread in densely populated regions, and therefore might be controlled by constant surveillance and early intervention [58]. This could also partly explain the relatively high rate of MRSA in Western Pacific and South-east Asia in our analysis.

MRSA BSI predictors among cancer patients were reported in three studies. All of the studies compared MRSA to MSSA, but each study contained different clinical factors, which precluded a pooled analysis. The identified predictors were mainly related to healthcare-associated infections and immunosuppression. In a study exploring MRSA BSI predictors in HIV-infected patients, multivariate analysis revealed that frequent hospitalization, low numbers of CD4+ peripheral cells, and previous administration of beta-lactams were found to be independent risk factors of MRSA development [59], which is partly in line with the findings in cancer patients. Prior antibiotic use was identified as the only independent predictor of MRSA bacteremia after analysis of data from two prospective multi-center studies in the general population [60]. Unfortunately, this clinical event frequently experienced by cancer patients was not studied in our included studies. Further studies comparing MRSA BSIs with all-cause BSIs and involving more demographic and clinical factors are needed to establish a reliable prediction rule for MRSA BSIs in the cancer population.

As reported in the previous researches, the 30-day mortality of MRSA BSIs ranged from 16 to 44% in general hospital populations [11, 20, 61–63]. This mortality was attributed to multiple complications of MRSA bacteremia, including infective endocarditis, deep tissue abscess, and septic shock [64]. However, data on the mortality rate of MRSA-BSIs in cancer patients is scarce, and hard to make a comparison. When it comes to the risk factors for mortality among patients with MRSA bacteremia, community-onset infection, secondary BSI, and vancomycin MIC≥2 g/mL were significant in cancer patients [53]. The relation of vancomycin MIC and mortality was also found in the non-cancer population [61, 65]. Notably, several studies have identified the association of inappropriate empirical antibiotic treatment with increased mortality among patients with MRSA BSIs [20, 62, 63, 66], and cancer patients were at an increased risk of receiving inappropriate therapy [62]. Appropriate initial antibiotic treatment should, therefore, gain adequate attention among patients with malignancy.

Several limitations of our study deserve consideration. First, all the included studies are observational, and there is a possibility of selection and observational bias. Second, the data are limited on BSIs solely in patients with solid malignancies as well as on BSIs from some geographic regions. Thus, we could not depict a more comprehensive picture of MRSA BSIs in cancer patients. Third, a direct comparison of the MRSA prevalence between cancer patients and general patients could not be achieved, which is precluded by the huge population of the latter group. Fourth, we did not find any study that provided incidence data after the search, so we could not predict the incidence trend. Fifth, a comparison between MRSA and MSSA was not made in our study, because we aimed to study MRSA which was a greater threat to life and the economy. Finally, the scarce information on predictor and mortality of MRSA bacteremia in cancer patients did not allow us to perform risk difference analysis and further establish decent prediction rules for MRSA development and mortality. Nevertheless, this study does provide relevant information on the prevalence, predictors, and mortality of bloodstream infections due to MRSA in the cancer population.

## Conclusions

The global prevalence of MRSA BSIs among cancer patients is relatively low. However, methicillin resistance was detected in nearly half of *S. aureus* isolated in the blood of cancer patients. Moreover, the rates of MRSA BSIs in patients with malignancy did not decline over time as MRSA BSIs in the general hospital population and the high mortality rate was related to MRSA BSIs in cancer patients, suggesting the severity of MRSA bacteremia among cancer patients. As the current information concerning MRSA bacteremia among the population of cancer is still limited, especially with regard to risk factors associated with MRSA development and mortality. Further researches are warranted to explore the predictors and risk factors for mortality of MRSA bacteremia in the population of cancer, as well as more effective infection control measures to decrease the rate of MRSA bacteremia.

## Supplementary Information

**Additional file 1: Figure S1.** Funnel plot

**Additional file 2: Table S1.** Quality assessment of included studies

**Additional file 3: Table S2.** Characteristics of studies included in the analysis of MRSA predictors and mortality

## Data Availability

The datasets used and/or analyzed during the current study are included in the manuscript.

## Abbreviations

MRSA: Methicillin-resistant *Staphylococcus aureus*
BSI: Bloodstream infection
SAB: *S. aureus* bacteremia
CI: Confidence interval
PRISMA: Preferred Reporting Items for Systematic Reviews and Meta-Analyses
WHO: World Health Organization
MSSA: Methicillin-susceptible *S. aureus*
CA-MRSA: Community-associated methicillin-resistant *Staphylococcus aureus*
