# Supplementary figures and images for: Prevalence, predictors, and mortality of bloodstream infections due to methicillin-resistant Staphylococcus aureus in patients with malignancy: systemic review and meta-analysis
Source: BMC Infect Dis. 2021 Jan 14;21:74. doi: 10.1186/s12879-021-05763-y (PMC7809798; doi:10.1186/s12879-021-05763-y)

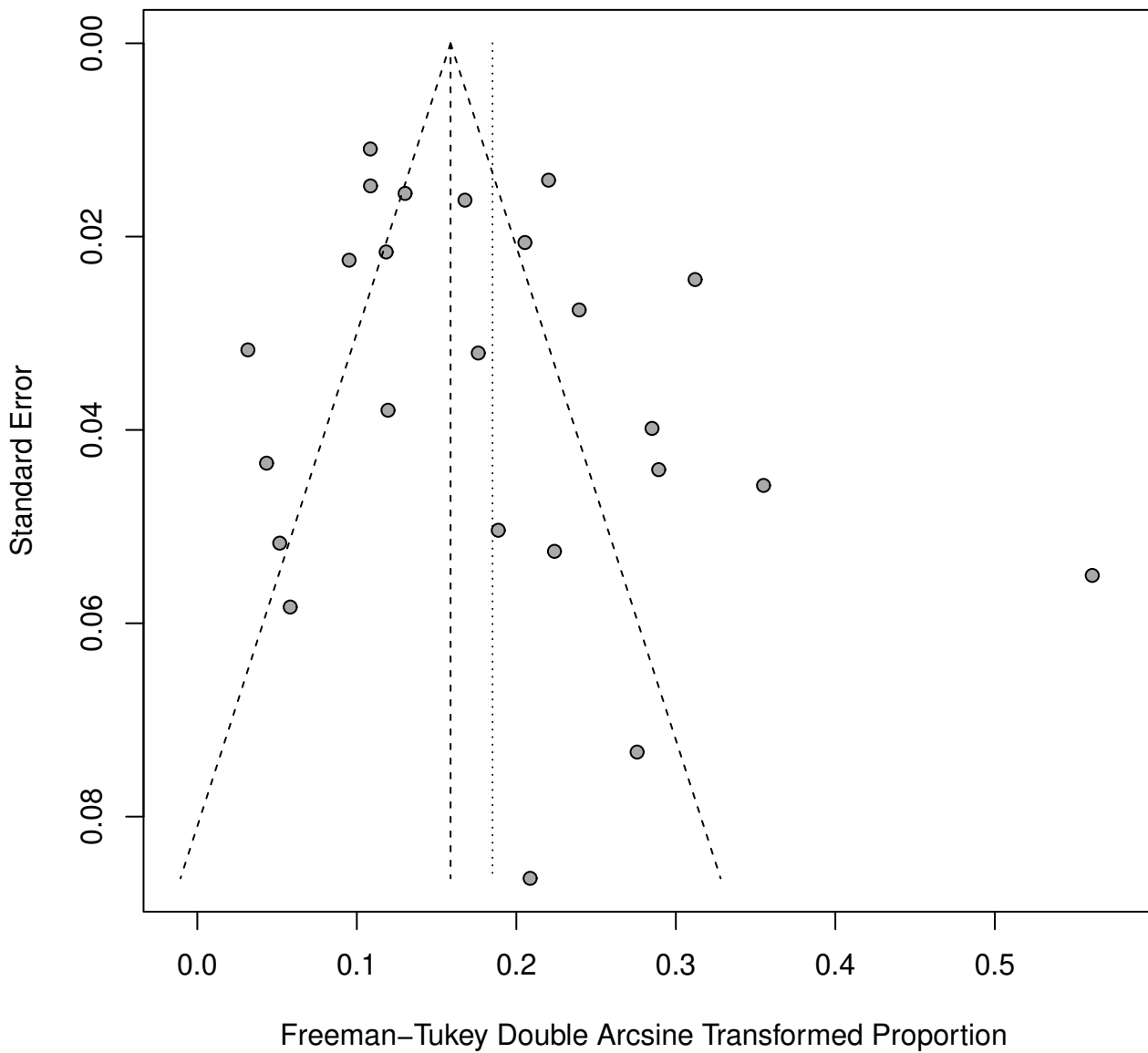

Supplement: Supplementary file 1 — Additional file 1: Figure S1. Funnel plot [file 12879_2021_5763_MOESM1_ESM.pdf]
