# Supplementary material for: Prevalence, predictors, and mortality of bloodstream infections due to methicillin-resistant Staphylococcus aureus in patients with malignancy: systemic review and meta-analysis
Source: BMC Infect Dis. 2021 Jan 14;21:74. doi: 10.1186/s12879-021-05763-y (PMC7809798; doi:10.1186/s12879-021-05763-y)
Supplement: Supplementary file 2 — Additional file 2: Table S1. Quality assessment of included studies [file 12879_2021_5763_MOESM2_ESM.docx]

**Supplementary Table S1**

Quality assessment of included studies

| Study | Selection | | | | Comparability | Outcome | | | Score |
| --- | --- | --- | --- | --- | --- | --- | --- | --- | --- |
|  | Representativeness of the exposed cohort | Selection of the non-exposed cohort | Ascertainment of exposure | Demonstration that outcome of interest was not present at start of study | Comparability of cohorts on the basis of the design or analysis | Assessment of outcome | Follow-up long enough for outcomes to occur | Adequacy of follow-up of cohorts |  |
| Anatoliotaki M | ★ | NA | ★ | NA | NA | ★ | ★ | ★ | 5 |
| Lai H-P | ★ | NA | ★ | NA | NA | ★ | ★ | ★ | 5 |
| El-Mahallawy H | ★ | NA | ★ | NA | NA | ★ | ★ | ★ | 5 |
| Greenberg D | ★ | NA | ★ | NA | NA | ★ | ★ | ★ | 5 |
| Velasco E | ★ | NA | ★ | NA | NA | ★ | ★ | ★ | 5 |
| Wang F-D | ★ | NA | ★ | NA | NA | ★ | ★ | ★ | 5 |
| Huang CC | ★ | NA | ★ | NA | NA | ★ | ★ | ★ | 5 |
| Schelenz S | ★ | NA | ★ | NA | NA | ★ | ★ | ★ | 5 |
| Baskaran ND | ★ | NA | ★ | NA | NA | ★ | ★ | ★ | 5 |
| Yamamoto M | ★ | NA | ★ | NA | NA | ★ | ★ | ★ | 5 |
| Miedema KGE | ★ | NA | ★ | NA | NA | ★ | ★ | ★ | 5 |
| Horasan ES | ★ | NA | ★ | NA | NA | ★ | ★ | ★ | 5 |
| Kara Ö | ★ | NA | ★ | NA | NA | ★ | ★ | ★ | 5 |
| Kang CI | ★ | NA | ★ | NA | NA | ★ | ★ | ★ | 5 |
| Kwon J-C | ★ | NA | ★ | NA | NA | ★ | ★ | ★ | 5 |
| Bodro M | ★ | NA | ★ | NA | NA | ★ | ★ | ★ | 5 |
| Chen CY | ★ | NA | ★ | NA | NA | ★ | ★ | ★ | 5 |
| Gedik H | ★ | NA | ★ | NA | NA | ★ | ★ | ★ | 5 |
| Arega B | ★ | NA | ★ | NA | NA | ★ | ★ | ★ | 5 |
| Obeng-Nkrumah N | ★ | NA | ★ | NA | NA | ★ | ★ | ★ | 5 |
| Bhat G | ★ | NA | ★ | NA | NA | ★ | ★ | ★ | 5 |
| Mvalo T | ★ | NA | ★ | NA | NA | ★ | ★ | ★ | 5 |
| Lubwama M | ★ | NA | ★ | NA | NA | ★ | ★ | ★ | 5 |
| Islas-Munoz B | ★ | NA | ★ | NA | NA | ★ | ★ | ★ | 5 |
| Srinivasan A | ★ | NA | ★ | ★ | NA | ★ | ★ | ★ | 6 |
| Bello-Chavolla OY | ★ | NA | ★ | ★ | NA | ★ | ★ | ★ | 6 |
| Mahajan SN | ★ | NA | ★ | ★ | NA | ★ | ★ | ★ | 6 |

NA, not applicable.
