# Supplementary material for: Prevalence, predictors, and mortality of bloodstream infections due to methicillin-resistant Staphylococcus aureus in patients with malignancy: systemic review and meta-analysis
Source: BMC Infect Dis. 2021 Jan 14;21:74. doi: 10.1186/s12879-021-05763-y (PMC7809798; doi:10.1186/s12879-021-05763-y)
Supplement: Supplementary file 3 — Additional file 3: Table S2. Characteristics of studies included in the analysis of MRSA predictors and mortality [file 12879_2021_5763_MOESM3_ESM.docx]

| Supplementary Table S1. Characteristics of studies included in the analysis of MRSA predictors and mortality | | | | | | | | |
| --- | --- | --- | --- | --- | --- | --- | --- | --- |
| Study | Country | Study design | Study period | Setting | No. of MRSA BSI cases | No. of MSSA BSI cases | | Analysis |
| Srinivasan et al. 2010 | USA | Single-center; retrospectively | 2000-2007 | children with ST or HM | 10 | | 42 | Predictors |
| Kang et al. 2012 | South Korea | Multi-center; prospectively | 2006-2007, 2008-2009 | adult with ST or HM | 59 | | 63 | Predictors |
| Bello-Chavolla et al. 2018 | Mexico | Single-center; retrospectively | 2006-2015 | adult with ST or HM | 95 | | 355 | Predictors; mortality |
| Mahajan et al. 2012 | USA | Single-center; retrospectively | 2001-2009 | adult and children with ST or HM | 223 | | NR | Mortality |
| MRSA, methicillin-resistant Staphylococcus aureus; BSI, bloodstream infection; MSSA, methicillin-susceptible Staphylococcus aureus; ST, solid tumors; HM, hematological malignancy; NR, not reported; | | | | | | | | |
